# The Influence of Polyamide-6 on the Structure and Functional Properties of Composite Hydrogels Based on Polyvinylpyrrolidone Copolymers

**DOI:** 10.3390/polym18192333

**Published:** 2026-09-24

**Authors:** Taras Hrytsenko, Nataliia Baran, Ludmila Dulebova, Jan Varga

**Affiliations:** 1 Department of Chemical Technology of Plastics Processing, Lviv Polytechnic National University, 12, St. Bandera Str., 79013 Lviv, Ukraine; taras.o.hrytsenko@lpnu.ua (T.H.); nataliia.m.baran@lpnu.ua (N.B.); 2 Department of Technology, Materials and Computer Supported Production, Faculty of Mechanical Engineering, Technical University of Košice, 74 Mäsiarska, 040 01 Košice, Slovakia; jan.varga@tuke.sk

**Keywords:** polyvinylpyrrolidone, 2-hydroxyethyl methacrylate, polymers, hydrogels, polyamide, interpolymer complex, modification, composites, strength

## Abstract

Controlling the balance between mechanical characteristics and sorption–diffusion properties remains one of the main challenges in the development of hydrogel materials. In this work, a method for obtaining composite copolymers of polyvinylpyrrolidone (PVP) with 2-hydroxyethyl methacrylate (HEMA) by modifying them with polyamide-6 (PA-6) during polymerization is proposed. PA-6/pHEMA-gr-PVP composite copolymers and hydrogels based on them were synthesized by polymerization of 2-hydroxyethyl methacrylate in the presence of a combined polymer matrix containing unbound PVP and a preformed PVP/PA-6 interpolymer complex (IPC). The existence of the IPC and its effect on the hydrogel structure were investigated using viscosity measurements, Fourier transform infrared spectroscopy, X-ray diffraction, and scanning electron microscopy. The results of the study show that PA-6 and PVP form a hydrogen-bonded IPC, which modifies the supramolecular organization of the hydrogel structure. This structural rearrangement increases the density of physical crosslinking within the polymer network, leading to improved mechanical strength and elasticity while reducing the water content, swelling rate, and osmotic permeability of the hydrogels. The obtained results demonstrate an effective approach to designing the structure of hydrogels and allow a simultaneous adjustment of their mechanical, sorption, and diffusion properties. This opens new possibilities for the development of mechanically strong hydrogel membranes with adjustable permeability for membrane and biomedical applications.

## 1. Introduction

Hydrogel materials are a class of three-dimensional polymeric materials with a cross-linked structure that are capable of absorbing and retaining significant amounts of water without losing the integrity of the polymer network [1,2]. Due to the combination of high hydrophilicity, biocompatibility and the ability to adjust physicochemical properties, hydrogels have found wide applications in tissue engineering [3,4], pharmaceuticals [5,6], membrane and film technologies [7,8], drug delivery systems [9,10], wound healing [11,12], various types of sensors [13,14], and soft robotics [15,16]. Recently, polymer hydrogels have attracted considerable attention due to their high water content and biocompatibility, leading to significant progress in their design, synthesis, and application as biological and biomedical materials [17,18,19,20,21].

Among hydrogel materials, polymer systems based on 2-hydroxyethyl methacrylate (HEMA) are widely used [22,23]. PolyHEMA and its copolymers are characterized by high transparency, biocompatibility, stability in aqueous media, and the ability to regulate their mechanical and sorption properties by changing the formula of the polymerization composition [24,25,26]. In particular, these properties have led to the widespread use of such materials in biomedical applications. Recent studies have demonstrated that modifying the structure of pHEMA-based hydrogels enables effective control over their porosity, swelling degree, and diffusion characteristics [27,28,29]. One effective approach to regulating the properties of pHEMA-based hydrogels is the incorporation of water-soluble polymers, in particular polyvinylpyrrolidone (PVP), into the polymer system [30]. PVP is characterized by high hydrophilicity, biological inertness, and the ability to form hydrogen bonds with various functional groups [31,32,33]. Copolymerization of HEMA with PVP promotes the formation of a hydrophilic polymer network [30,34,35] with increased sorption capacity and improved diffusion characteristics [30,34,35,36,37]. It has been established that the formation of specific intermolecular complexes is possible in such systems, which can affect the kinetics of polymerization and the structure of the formed hydrogel. In particular, recent studies have shown that the interaction between the HEMA monomer and PVP macromolecules leads to the formation of charge-transfer complexes, which accelerates polymerization and promotes the formation of a more homogeneous polymer structure [30,34,36].

At the same time, one of the main disadvantages of hydrogel materials, particularly those based on HEMA/PVP copolymers (pHEMA-gr-PVP), is their relatively low mechanical strength [38,39,40]. This significantly limits their use in applications involving mechanical loads or pressure gradients, such as medical films [41], blood vessel prostheses [42], and cosmetic masks [43]. In this regard, considerable attention has been devoted to the development of composite hydrogel materials in which the polymer matrix is modified by incorporating various functional components or fillers [44,45,46,47]. The use of modifiers of different types or the formation of interpolymer complexes (IPCs) can significantly improve the mechanical properties of hydrogels without substantially compromising their sorption properties [48,49]. Modification can be performed both at the surface by using reinforcement [50] or forming multilayer materials [39,45], as well as in the bulk of the material [51]. For example, the reinforcement with a polypropylene network provides hydrogels with increased strength, stiffness, and dimensional stability, while also improving their thermal resistance [50]. However, due to the structural heterogeneity of reinforced materials, they might be susceptible to the formation of internal defects and microcracks under external influences. The physicomechanical characteristics of reinforced hydrogels can be improved by establishing chemical bonds between the polymer matrix and the reinforcing material [50,52].

Multilayer composite hydrogels are obtained either by applying a hydrogel layer onto materials of different nature [53,54] or by applying a modifying layer onto the hydrogel surface [39,55].

The proposed method in [39] for obtaining composite hydrogel films based on pHEMA-gr-PVP copolymers involves two stages: the preparation of a hydrogel film substrate and the formation of an ultrathin polymer reinforcing layer on its surface. A PA-6/PVP modifying solution in formic acid was used to obtain the reinforcing layer.

However, the limitation of the developed method from a technological point of view lies in its laboriousness, multistage and long duration. At the same time, from a practical perspective, it is difficult to obtain a uniform reinforcing coating of consistent thickness with this method, especially when the hydrogel product has a developed surface.

One promising approach to modifying hydrogels based on pHEMA-gr-PVP copolymers is the incorporation of polyamide into the polymer matrix. Due to the presence of amide groups, polyamide macromolecules can form hydrogen bonds with hydrophilic polymer systems [51,56], which may promote the formation of structurally stable polymer compositions.

Therefore, there is a need to move from local surface modification toward the formation of a polyamide-containing phase directly within the bulk of the polymerization system, whereby modification of the hydrogel matrix is carried out directly during copolymer synthesis (bulk modification), prior to the formation of the polymer network. It is assumed that hydrogen bonds between the amide groups of PA-6 and the carbonyl groups of PVP will promote the formation of additional physical network junctions, thereby altering its supramolecular organization. In contrast to surface reinforcement, this approach involves the distribution of the modifying polyamide phase throughout the entire hydrogel volume. This is particularly important for hydrogel membranes, since surface modification primarily alters the properties of the near-surface layer, whereas bulk incorporation of the modifier can simultaneously affect the structure, mechanical strength, sorption, and mass transport throughout the material.

Thus, the scientific novelty of this work lies in establishing the patterns of structural and functional changes in the pHEMA-gr-PVP hydrogel matrix upon its bulk modification with a preformed PA-6/PVP interpolymer complex (IPC). Particular attention is given to elucidating the interactions between PA-6 and PVP and to assessing the effect of modification on the mechanical, sorption, and diffusion properties of hydrogel materials based on pHEMA-gr-PVP copolymers.

The aim of this work is to investigate the effect of bulk modification of pHEMA-gr-PVP copolymers by a polyamide phase on their structure, physicomechanical, sorption, and diffusion characteristics.

## 2. Materials and Methods

### 2.1. Materials

To obtain modified polyamide copolymers pHEMA-gr-PVP (PA-6/pHEMA-gr-PVP), the following materials were used: 2-hydroxyethyl methacrylate (Sigma Chemical Co, Saint Louis, MO, USA), purified and distilled in a vacuum (residual pressure = 130 N/m^2^, T_boil_ = 351 K); highly purified polyvinylpyrrolidone (MW = 12 × 10^3^ g/mol; AppliChem GmbH, Darmstadt, Germany), dried at 338 K in a vacuum for 2–3 h before use; polyamide PA-6 “Tarnamid-27” (Grupa Azoty S.A., Tarnów, Poland); formic acid (Poch S.A., Gliwice, Poland), marking “h”; iron (II) sulfate of analytical grade (p.a.); and distilled water. Generative Artificial Intelligence tools, specifically ChatGPT (OpenAI, GPT-5.6 Luna), were used in Scheme 1 and Figure 2, which were obtained and generated in July 2026 (Scheme 1) and September 2026 (Figure 2).

### 2.2. Method for Preparing PA-Modified Hydrogels

The initial compositions are characterized by high fluidity, which enables their processing in molds by casting and rotational molding [41]. In this work, composite hydrogel films were prepared in a polymerization mold, the cavity of which was formed by two flat glass surfaces. The procedure for obtaining the modified hydrogel films is shown in Scheme 1; the compositions used in the study are presented in Table 1. The procedure for preparing composite hydrogel films is as follows: In the first stage, PA-6 is dissolved in HCOOH with the addition of distilled water (1/2 of the required amount).

Part of the required amount of PVP is then added to the resulting solution under stirring to form the interpolymer complex PA-6/PVP (PA-6:PVP = 95:5 wt.%; IPC:HCOOH = 7:93 wt.%). The optimal composition of the IPC and the IPC:HCOOH ratio was established in previous studies [39,51,55,57]. After the complete dissolution of PVP, HEMA is introduced. In a separate vessel, the catalyst FeSO_4_ (0.1 wt.% of the PMC mass (Table 1)) is dissolved in water (1/2 of the required amount), followed by the addition of the remaining PVP (stage II). After obtaining two homogeneous solutions, they are combined and thoroughly mixed (stage III). The resulting reaction mixture is dispensed into a pre-prepared mold and polymerized at 20–25 °C, combining the molding and polymerization stages of the reaction mixture (stage IV). After mixing the two solutions, the resulting reaction mixture consists of HEMA/CM/S, where CM denotes the combined polymer matrix (PVP + IPC) and S represents the solvent (H_2_O + HCOOH). The polymerization stage lasts 60–90 min, depending on the HEMA:CM ratio in the reaction composition. After the completion of the polymerization stage, the mold is placed in a water bath for 1 h (stage V). The mold is then opened, and the finished film is released (stage VI). To completely remove formic acid, the film is washed in distilled water for 24 h (stage VII), followed by hydrothermal treatment at 50 °C for 30 min (stage VIII). The resulting films were obtained with a thickness of 0.5 ± 0.05 mm.

### 2.3. Measurements and Characterization

#### 2.3.1. Standard Methods of Instrumental Research

X-ray diffraction (XRD) analysis was carried out using an Aeris Research benchtop powder X-ray diffractometer (Malvern Panalytical B.V., Almelo, the Netherlands) equipped with a Cu Kα radiation source (λ = 1.5406 Å). The diffraction patterns were recorded over a 2θ range of 6–60° at an operating voltage of 40 kV and a tube current of 15 mA. The diffraction data were collected with a step size of 0.0217°.

Fourier transform infrared (FTIR) spectra were recorded using a Spectrum Two FTIR spectrometer (PerkinElmer Inc., Waltham, MA, USA) equipped with a universal attenuated total reflectance (UATR) accessory. The spectra were collected over a wavenumber range of 4000–400 cm^−1^ with a spectral resolution of 4 cm^−1^ by averaging 24 scans for each sample.

The morphology of composite hydrogels was investigated using a Thermo Scientific Apreo 2 scanning electron microscope (Thermo Fisher Scientific, Waltham, MA, USA). Prior to imaging, the hydrogel samples were freeze-dried, fractured to expose their internal structure, mounted on aluminum stubs using conductive carbon tape, and sputter-coated with a thin gold layer to minimize charging effects. SEM images were acquired in secondary electron (SE) mode at an accelerating voltage of 5 kV and a working distance of approximately 8–10 mm.

#### 2.3.2. Viscosity Measurement of Solutions

The dynamic viscosity of the solutions was measured according to the method described in ISO 3219: “Plastics—Polymers/resins in the liquid state or as emulsions or dispersions—Determination of viscosity using a rotational viscometer with defined shear rate” [58]. Dynamic viscosity was measured using a Rheostress RS75 rheometer (Haake, Thermo Electron GmbH, Karlsruhe, Germany) at a constant temperature of 20 ± 0.1 °C and a shear rate of 50 s^−1^. Measurements were taken using a coaxial cylinder measuring system (Couette geometry DG 25).

The specific viscosity of the polymer solutions was measured using an Ubbelohde suspended-level capillary viscometer immersed in a thermostatically controlled water bath maintained at 25.0 ± 0.1 °C. A series of dilute polymer solutions was prepared using a formic acid/water solvent mixture. Measurements were performed according to ISO 1628-1:2024 [59].

#### 2.3.3. Puncture Strength and Relative Elongation at Puncture

The puncture strength (ϭ, MPa) and relative elongation at puncture (ɛ, %) of the synthesized materials were determined using a tensile testing method. Film samples were fixed in a ring-shaped clip and punctured with a pin indenter using a tensile testing machine “Kimura” 050/RT-601U (Kimura Machinery, Ishinomaki, Japan) with the sample strain rate of 25 mm/min. The puncture strength and relative elongation at puncture of the hydrated films were calculated according to Equations (2) and (3), respectively:

(1) $$\sigma =\frac{F}{D\times h}\times 10^{-6}$$

(2) $$\varepsilon =257\times \frac{l}{D}-25$$

where F is the force at which the sample ruptures, N; D is the diameter of ring-shaped clamp hole, m; h is the film thickness, m; and l is the pin displacement from the moment of contact with the sample to the moment of rupture, m (Figure 1).

#### 2.3.4. Boundary Water Absorption

Boundary water absorption (W, wt.%) was determined gravimetrically as the difference between the masses of the dry (m_0_, g) and swollen (m_1_, g) samples:

(3) $$W=\frac{m_{1}-m_{0}}{m_{1}}\times 100\%$$

#### 2.3.5. Swelling Kinetics

The swelling kinetics were characterized by the degree of water absorption α, expressed in g(H_2_O)/g(polymer). The value of α at time t was determined as the ratio of the mass of liquid absorbed by the polymer during swelling (m_t_) to the mass of the dry polymer (m_0_):

(4) $$\alpha_{t}=(m_{t}-m_{0})/m_{0}$$

#### 2.3.6. Permeability of Hydrogel Films

The permeability of the synthesized hydrogel films was measured without applying external pressure, using the osmosis method [57]. A 4% aqueous solution of sodium chloride was used as a model solution. To determine osmotic permeability, an osmometer (Figure 2) was used. The device was placed in a vessel containing distilled water and filled with a salt solution. The amount of solution that passed through the film was determined from the change in the solution level in capillary 3. The amount of salt that permeated through the investigated film was determined from its concentration in vessel 5 using a conductivity meter MP-513 (Ulab, Shanghai, China). Pre-calibration of device was performed by salt water solutions of known concentrations and a calibration graph of resistance/salt concentration dependence was obtained. By measuring the conductivity of aqueous solution and using the calibration graph, the amount of salt that had penetrated through the membrane was determined. The ratio G/S was calculated, where G is the amount of salt that penetrated through the film by a certain time (mol), while S is the effective area of the film (m^2^).

## 3. Results

### 3.1. Intermolecular Interactions in the Reaction Mixture

The developed method for modifying pHEMA-gr-PVP copolymers and synthesizing composite hydrogel materials based on them is enabled by the high reactivity of the HEMA/PVP polymer–monomer composition (PMC), as well as by the possibility of conducting polymerization in a solvent medium [30,36]. Previous work has shown that the high reactivity of HEMA/PVP compositions is associated with the so-called “matrix effect”, which involves the solvation of monomer molecules within the PVP polymer matrix (Scheme 2a) and the formation of a charge-transfer complex (CTC) [30,34].

The use of small amounts of active additives, in particular salts of metals with variable oxidation states, in the initial composition initiates the formation of a ternary CTC (Scheme 2b). Complexation among HEMA, PVP, and metal ions significantly affects the polymerization kinetics and the formation of the polymer structure during the process of hydrogel synthesis [35,60]. Compositions based on HEMA/PVP in the presence of transition-metal salts, in particular iron (II) sulfate, polymerize rapidly at room temperature in air [34,35,36].

Considering the complex-forming abilities of PVP and PA-6, an interaction between the polymers can be expected. Certain differences can already be observed from the appearance of the solutions (Figure 3). The PVP solution in the water/formic acid system is transparent and light brown, whereas the PA-6 solution is transparent and colorless. In contrast to these, the PA-6/PVP mixture appears light brown and turbid.

The light-brown color of the PVP solution may indicate weak donor–acceptor interactions in the PVP/formic acid system. The appearance of turbidity after mixing PA-6 and PVP indicates a change in the state of the polymer system and may be associated with the formation of associative structures or a change in the solubility of the components. However, visual observations alone are insufficient to unambiguously determine the nature of these changes.

To verify the interaction between PVP and PA-6, a comprehensive approach combining viscosity analysis of concentrated and dilute solutions with FTIR spectroscopy was employed.

It was found that with the increasing total concentration of the PA-6/PVP polymer mixture in the water/formic acid solvent, the dynamic viscosity of the concentrated solutions increases systematically (Figure 4a). This is associated with an increase in the number of macromolecules in the system and interchain interactions. At the same time, it was found that with an increase in the PVP content in the PA-6/PVP mixture, the viscosity of the solutions decreases. This change in viscosity indicates specific interactions between the components that are characteristic of IPC formation [61,62]. Moreover, in more concentrated solutions, the decrease in viscosity with increasing PVP content is more pronounced compared with that of pure PA-6 solutions.

In solutions, polyamide forms associated structures through intermolecular hydrogen bonds (–N-H···O=C–). The introduction of PVP leads to a redistribution of these interactions, since the carbonyl groups of PVP act as hydrogen-bond acceptors. Consequently, some of the PA-6–PA-6 bonds are replaced by PA-6–PVP interactions. This is accompanied by a transition of macromolecular chains to a more extended conformational state, which reduces the effective hydrodynamic volume of polymer associates in solution. In dilute solutions of the PA-6/PVP polymer mixture (C_PA-6/PVP_ = 0.5 wt.%), the specific viscosity of the solutions decreases compared with that of pure PA-6, reaching a minimum at a PVP content in the mixture of up to 2 wt.%, followed by a slight increase (Figure 4b). This effect is observed at different formic acid concentrations in the HCOOH/H_2_O system. A decrease in the reduced or dynamic viscosity of the solution can be considered indirect evidence of IPC formation [61,62].

To investigate the intermolecular interactions in the PA-6/PVP system and clarify their contribution to the formation of the composite hydrogel network, the FTIR spectra of separately dried PVP, PA-6, and a PA-6/PVP blend prepared from a water/formic acid solution were analyzed (Figure 5).

The FTIR spectrum of PVP exhibits characteristic absorption bands corresponding to the pyrrolidone ring. The intense absorption band in the 1650–1670 cm^−1^ region is associated with the stretching vibrations of the carbonyl group (ν(C=O)), while the bands at 2950–2850 cm^−1^ originate from the asymmetric and symmetric stretching vibrations of the methylene groups [63,64,65]. The absorption band at 1280–1290 cm^−1^ is associated with the stretching vibrations of the C–N lactam ring. In addition, the infrared spectrum of PVP clearly shows a band at 1717 cm^−1^, which is attributed to the stretching vibrations of the carbonyl group [64,65]. The spectrum of PA-6 is characterized by typical absorption bands of amides [66]. The broad band centered at approximately 3290 cm^−1^ corresponds to N–H stretching vibrations. The absorption bands at approximately 2935 and 2860 cm^−1^ are attributed to CH_2_ stretching vibrations, while the characteristic bands at approximately 1633 and 1537 cm^−1^ correspond to Amide I and Amide II vibrations, respectively [56,67,68]. These bands are highly sensitive to changes in hydrogen bonding within the polyamide structure.

Comparison of the spectra of the individual polymers with that of the PVP/PA-6 blend reveals significant changes in the amide–carbonyl region (1500–1700 cm^−1^). The characteristic carbonyl band of PVP loses its distinct profile and merges with the polyamide band at 1633 cm^−1^, resulting in a single band at 1636 cm^−1^. The band at 1717 cm^−1^, assigned to the carbonyl group of PVP, also disappears. These changes in the spectral profile indicate a change in the energy state of the carbonyl groups of PVP due to their participation in intermolecular hydrogen bonds. At the same time, the position of the stretching vibration bands of methylene groups (2935–2865 cm^−1^) in the IR spectrum of PA-6 remains practically unchanged, indicating the absence of significant changes in the structure of aliphatic fragments of polymer chains. Therefore, the interaction is localized mainly in the region of polar functional groups. Changes in the positions and profiles of the polyamide bands at 1537 and 3290 cm^−1^ are also observed, with shifts to 1540 and 3297 cm^−1^, respectively. These spectral changes indicate changes in the local electronic environment of both carbonyl and amide groups and are consistent with the formation of specific intermolecular hydrogen bonds. The observed interaction can be attributed to hydrogen bonding between the carbonyl oxygen atom of the pyrrolidone ring and the hydrogen atom of the amide group of PA-6 (C=O···H–N). Since no additional absorption bands corresponding to new covalent bonds are observed, the interaction can be considered as physical in nature. Thus, the results obtained are consistent with the formation of an IPC stabilized by a hydrogen bond network.

### 3.2. Effect of PA-6 on the Structure of pHEMA-gr-PVP Copolymers

To evaluate the effect of the PA-6/PVP IPC on the supramolecular organization of the composite hydrogels, X-ray diffraction analysis was performed on samples containing different amounts of the IPC in the polymer composition (Figure 6): Sample 1—PVP:IPC = 95:5 wt.%; Sample 2—PVP:IPC = 70:30 wt.%.

The X-ray diffraction patterns of both composites do not show intense, narrow diffraction peaks characteristic of highly crystalline polyamide. However, broad diffuse peaks are observed in the regions of 19–23°, 30–32°, and 40–44°, which is characteristic of a predominantly amorphous polymer matrix. At a low PA-6 content (curve 1), the characteristic peaks of PA-6 are significantly broadened, partially overlap with the amorphous halo of the pHEMA-gr-PVP polymer matrix [39], and do not exhibit the characteristic structure of the α-phase. In this case, during hydrogel formation, the PA-6 chains interact with the carbonyl groups of pHEMA and PVP, resulting in the formation of intermolecular hydrogen bonds. Consequently, the regular packing of PA-6 macromolecules is disturbed, leading to a decrease in the degree of polyamide crystallinity. This diffraction pattern indicates that, after polymerization, the compositions form a predominantly amorphous, spatially crosslinked system in which long-range crystalline order is practically absent.

With increasing PA-6 content (curve 2), the overall shape of the diffraction pattern changes only slightly. However, a maximum appears at approximately 23.8°. The appearance of this maximum is noteworthy, since the 23.6–24° region is one of the characteristic regions of the α-phase of PA-6 [69,70]. This observation may indicate the gradual formation of more ordered domains of the polyamide component, although it does not indicate the formation of a fully developed crystalline α-phase of PA-6. Thus, an increase in the concentration of the IPC may contribute to the formation of locally ordered regions of polyamide.

Particular attention should also be paid to the 19–23° region. For the composite with a PVP:IPC ratio of 95:5, the amorphous halo of pHEMA-gr-PVP, the PA-6 reflections, and the effects of PVP–PA-6 hydrogen bonding are simultaneously superimposed in this region. As a result, the maximum does not exhibit the typical PA-6 peak. This may indicate that a part of the PA-6 chains does not crystallize as pure polyamide, and the crystallinity of the polyamide does not increase. With an approximately sixfold increase in the PA-6 content of the copolymer (Figure 6, curve 2), a certain increase in its crystalline reflections (19.2° and 23.8°) is observed. Since the intensities of these reflections remain low, it can be concluded that the polyamide chains transition to a more locally ordered state, while hydrogen bonding with PVP prevents the formation of large crystallites. Thus, the polyamide does not form a separate crystalline phase but remains incorporated into the IPC with PVP, which is consistent with the IR spectroscopy data. The obtained results show that the IPC formation alters the supramolecular organization of the polyamide, reducing the long-range crystalline order while promoting the formation of locally ordered domains.

Given these findings, it can be concluded that the main consequence of introducing the IPC is not the crystallization of polyamide, but rather the modification of the supramolecular organization of the copolymer matrix, which, in turn, determines the structure, and therefore the properties of the synthesized hydrogels.

SEM analysis demonstrated that the morphology of the modified pHEMA-gr-PVP copolymers depends on the content of the PA-6/PVP IPC. A homogeneous, smooth surface is characteristic of the sample with the lowest IPC content (PVP:IPC = 95:5) (Figure 7a). The fracture surface (Figure 7b) exhibited a predominantly plate-like morphology with relatively smooth fracture planes and only a few secondary defects. Examination of the cross-sectional surface (Figure 7c) revealed no evidence of phase separation, microvoids, or pronounced microstructural heterogeneity. With an increase in the IPC content to a PVP:IPC ratio = 70:30, the microrelief develops, the surface roughness increases, and the morphology of the fracture surface becomes more complex, which is manifested in the appearance of local inhomogeneities and disruption of the regularity of the lamellar relief (Figure 7d–f). Local bridges appear between the layers, which may indicate the stronger cohesion of the material. At the same time, the absence of distinct agglomerates and signs of interfacial delamination is consistent with a relatively homogeneous composite morphology at the scale accessible by SEM.

### 3.3. Properties of Composite Hydrogels Based on PA-6/pHEMA-gr-PVP Copolymers

Among the main characteristics that determine the suitability of hydrogel materials for particular applications are strength, elasticity, swelling capacity in solvents (particularly water), and diffusion–transport properties.

The strength of the hydrogel film samples was characterized by their puncture strength (ϭ, MPa), while elasticity was evaluated by the relative elongation at puncture (ɛ, %). Figure 8 presents the study results as a function of the PMC and IPC contents. An increase in the PVP content in the initial composition leads to a decrease in strength, which is consistent with the results of previous studies on pHEMA-gr-PVP copolymers [34,36]. The observed effect is associated with a decrease in the crosslinking density of the polymer network and the plasticizing effect of PVP [30,34,36]. At the same time, the introduction of PA-6 contributes to an increase in strength due to the formation of additional physical network junctions [71] and reinforcement of the polymer structure. PA-6 is a relatively rigid crystalline polymer that acts as a reinforcing component. The total effect is determined by the ratio of the components in the PMC and the extent of IPC formation in the system.

In order to combine the stages of obtaining hydrophilic polymers and their subsequent swelling, the synthesis of modified hydrogels was carried out in the presence of a solvent—water. In this regard, the dependence of the properties of the obtained composite hydrogels on the amount of solvent in the initial composition was studied (Figure 9). In all cases, dilution of the composition with the solvent resulted in a decrease in the puncture strength of the samples in the swollen state (Figure 9a). At the same time, increasing the solvent content in the composition enhanced the elasticity of the composites, as evidenced by an increase in elongation at puncture.

The amount of solvent in the initial composition is an important factor determining the structure of the hydrogel materials, as it affects the degree of crosslinking of the polymer network [36]. An increase in the water content of the initial composition causes an increase in the molecular weight between cross-links in the polymer network (M_C_) and, consequently, leads to the formation of a polymer network with a lower crosslinking density [36], which is the main factor responsible for the loss of strength.

The increase in relative elongation at puncture with increasing solvent content in the polymer–monomer composition is also attributed to its effect on the structure of the copolymer. The introduction of the solvent into the composition results in the formation of a network with increased sizes of chain fragments between the crosslinking junctions [36] and with a greater ability to undergo conformational changes. In addition, the lower crosslinking density of the polymer network reduces spatial constraints to conformational changes. Therefore, under the load, longer chain segments between the crosslinking junctions are able to undergo greater extension than short ones, contributing to an increase in ε. At the same time, a comparative analysis of the properties of unmodified hydrogels [34,35] and the developed modified hydrogels demonstrated that bulk modification of the hydrogels with polyamide improves their mechanical strength while simultaneously enhancing their elasticity. For example, unmodified hydrogel materials based on pHEMA-gr-PVP copolymers, obtained from compositions with HEMA:PVP:H_2_O = 80:20:100 mass parts, exhibited a puncture strength of ϭ = 0.4 MPa and a relative elongation at puncture of ɛ = 235% [36,37], compared with ϭ = 0.72 MPa and ɛ = 335% for the modified hydrogels (Figure 9a).

The ability to swell in solvents is a key characteristic of a hydrogel, since it largely determines its mechanical and functional properties, in particular elasticity, biocompatibility, and permeability to low-molecular-weight compounds. Copolymers based on pHEMA-gr-PVP possess a developed porous morphology [30,34,36] and contain polar hydrophilic fragments in their structure, including hydroxyl and carbonyl groups of HEMA, as well as carbonyl groups of PVP (Scheme 2). The content of these functional groups, together with the level of porosity of the hydrogel matrix, directly affects the sorption properties of the material. The presence of pores contributes to the intensive solvent absorption due to capillary effects, which, in turn, affects the swelling process of the copolymer. The swelling capacity was evaluated based on the boundary water absorption factor (W, %). Previous works [30,34,36] established that an increase in the PVP content in PMC leads to an increase in the water content of the hydrogels. This is explained, on one hand, by the increase in the hydrophilicity of the polymer network due to the incorporation of chemically bonded PVP chains, and, on the other hand, by the increase in the free volume between macromolecules, resulting from the partial leaching of ungrafted PVP [34,36]. In addition, changes in the composition of PMC affect the parameters of the spatial structure of the copolymer: with an increase in PVP content, the crosslinking density of the polymer network decreases [34,36]. At the same time, the introduction of PA-6 into the structure of pHEMA-gr-PVP copolymers reduces their water absorption capacity (Figure 9b). It was established that the nature of the change in the water content of modified hydrogels with an increase in the PVP content in the initial composition differs from that of the unmodified copolymers. The greatest decrease in water content is observed for the PMC with a HEMA:CM ratio of 70:30 wt.%. (Figure 10, curve 3), whereas the smallest decrease is observed for the PMC with a HEMA:CM = 90:10 mass parts (Figure 10, curve 1). It is evident that the main role in reducing the water absorption capacity of modified pHEMA-gr-PVP copolymers is played by the presence of PA-6 in their structure and the formation of an IPC of PA-6 with PVP. This is primarily associated with the formation of additional physical junctions in the polymer network through hydrogen bonding between PA-6 with PVP and HEMA. The additional physical network increases the crosslinking density and reduces the mobility of molecular chains [72]. In hydrogels, hydrogen bonds can improve cohesion, acting as crosslinking domains [73]. During the formation of the IPC between PVP and PA-6, the hydrophilic centers of PVP become “blocked”. Whereas PVP is actively hydrated in the unmodified copolymer, upon the addition of PA-6, some of the hydrophilic centers of PVP become part of the IPC and are inaccessible to water. As a result, the number of water sorption centers decreases. Thus, the most pronounced reduction in water content is observed for systems with high contents of PVP and PA-6, where the intensity of IPC formation is the greatest. It should also be taken into account that the introduction of hydrophobic fragments of polyamide (hydrophobic methylene segments) into the hydrogel structure also reduces the overall hydrophilicity of the modified copolymer system. As expected, as in the case of unmodified pHEMA-gr-PVP copolymers, an increase in the solvent content in the initial composition leads to an increase in its content within the volume of the copolymer, which affects the increase in Mc and, consequently, improves the sorption capacity [36]. However, the water content of the modified hydrogels, due to the influence of PA-6, is somewhat lower than that of the unmodified ones (Figure 9b).

Since hydrogels are generally used in their swollen state, it is essential to determine the time required for the material to reach swelling equilibrium. Figure 11 presents the swelling kinetics of PA-6/pHEMA-gr-PVP copolymers as a function of PVP:IPC (a) and HEMA:CM (b).

To compare the effect of PA-6 on the swelling kinetics, the PMC with the highest PVP content (Figure 11a) and the composition with the highest PA-6 content in the IPC (Figure 11b) were selected, since these systems most clearly demonstrate the effect of PA-6, as part of the PVP/PA-6 complex, on the sorption capacity of modified copolymers. The introduction of polyamide in the form of a PA-6/PVP IPC leads to a significant change in the swelling kinetics of hydrogels based on pHEMA-gr-PVP copolymers (Figure 11a). In particular, a decrease in the water sorption rate is observed with increasing PA-6 content in the hydrogel, especially at the initial stage of the process, i.e., during the first 5 h. As can be seen from the presented kinetic curves, the formula of the initial HEMA:CM composition significantly affects both the swelling rate and the mechanism of water transport within the hydrogel volume (Figure 11b). For the system with the maximum PVP content (HEMA:CM = 70:30 mass parts), the lowest swelling rate is observed (Figure 11b, curve 3). The initial section of curve 3 is characterized by a slight slope, indicating limited water penetration into the polymer network. This behavior is attributed to the highest concentration of the PA-6/PVP IPC, which forms additional physical network junctions through intermacromolecular hydrogen bonding. As a result, the effective crosslinking density increases and the mobility of macrochains decreases, limiting both water diffusion and the relaxation of the polymer structure. For the system with the minimum PVP content (HEMA:CM = 90:10 mass parts), swelling occurs at the highest rate (Figure 11b, curve 1). The kinetic curve shows a steep initial rise, which can be attributed to the low IPC content, resulting in a less constrained polymer network with greater free volume and higher macrochain mobility. The obtained results are consistent with the formation of an additional physical network through the PA-6/PVP IPC, which governs the swelling behavior of the resulting hydrogels.

For medical applications, the permeability of hydrogels to aqueous solutions of salts and pharmacologically active compounds is also particularly important. The permeability of the obtained modified hydrogel films was evaluated by investigating the osmotic transport of sodium chloride as a model solute from an aqueous medium. It was found that the introduction of PA-6 as a component of the PA-6/PVP IPC into the pHEMA-gr-PVP copolymer structure leads to a decrease in the osmotic permeability of the hydrogel films (Figure 12a), despite their relatively high water content (Figure 10).

This decrease is attributed to the compaction of the polymer network due to the formation of additional physical junctions and a reduction in free volume. At the same time, PA-6 can form “diffusion barriers” in the form of local dense domains and regions of reduced mobility, resulting in increased structural heterogeneity and, consequently, reduced permeability. Under these conditions, the determining factor is not the sorption capacity, but the diffusion coefficient, which decreases significantly with increasing polyamide content. With increasing PVP content in the composition at a high polyamide content, a decrease in permeability is observed, which is associated with enhanced formation of the PA-6/PVP IPC and the corresponding densification of the hydrogel structure.

## 4. Conclusions

A method for the synthesis of composite hydrogels based on pHEMA-gr-PVP copolymers modified with PA-6 through the preliminary formation of the PA-6/PVP IPC in the reaction composition has been developed. The proposed approach ensures the uniform incorporation of PA-6 throughout the polymer matrix and the formation of structurally homogeneous hydrogel materials.

Comprehensive viscosity measurements of polymer solutions and FTIR spectroscopy confirmed the formation of an IPC between PA-6 and PVP, stabilized by a network of intermolecular hydrogen bonds. The formation of the IPC is accompanied by a rearrangement of interchain interactions in the polyamide, changes in the conformational state of the macromolecules, and a redistribution of hydrogen bonds within the system.

According to the XRD results, the introduction of PA-6 does not lead to the formation of a separate highly crystalline phase but changes the supramolecular organization of the composite. With increasing IPC content, locally ordered polyamide domains are formed while the predominantly amorphous structure of the hydrogel is maintained. SEM analysis confirmed the uniform distribution of the polyamide component without evidence of macrophase separation.

It was shown that the IPC formation affects the functional properties of hydrogels. The introduction of PA-6 increases the strength and elasticity of the materials due to the formation of additional physical junctions in the polymer network. At the same time, a decrease in the equilibrium water content, a slowdown in swelling kinetics, and a decrease in osmotic permeability are observed. These changes are associated with an increase in the effective density of physical crosslinking, a blockage of some of the hydrophilic centers of PVP, and a decrease in the free volume of the polymer matrix.

The results obtained indicate that regulating the extent of PA-6/PVP IPC formation is an effective tool for controlling the structure and the combination of mechanical, sorption, and diffusion properties of pHEMA-gr-PVP-based hydrogels, providing prospects for their use as functional membrane and biomedical materials.

## Figures and Tables

**Scheme 1:**
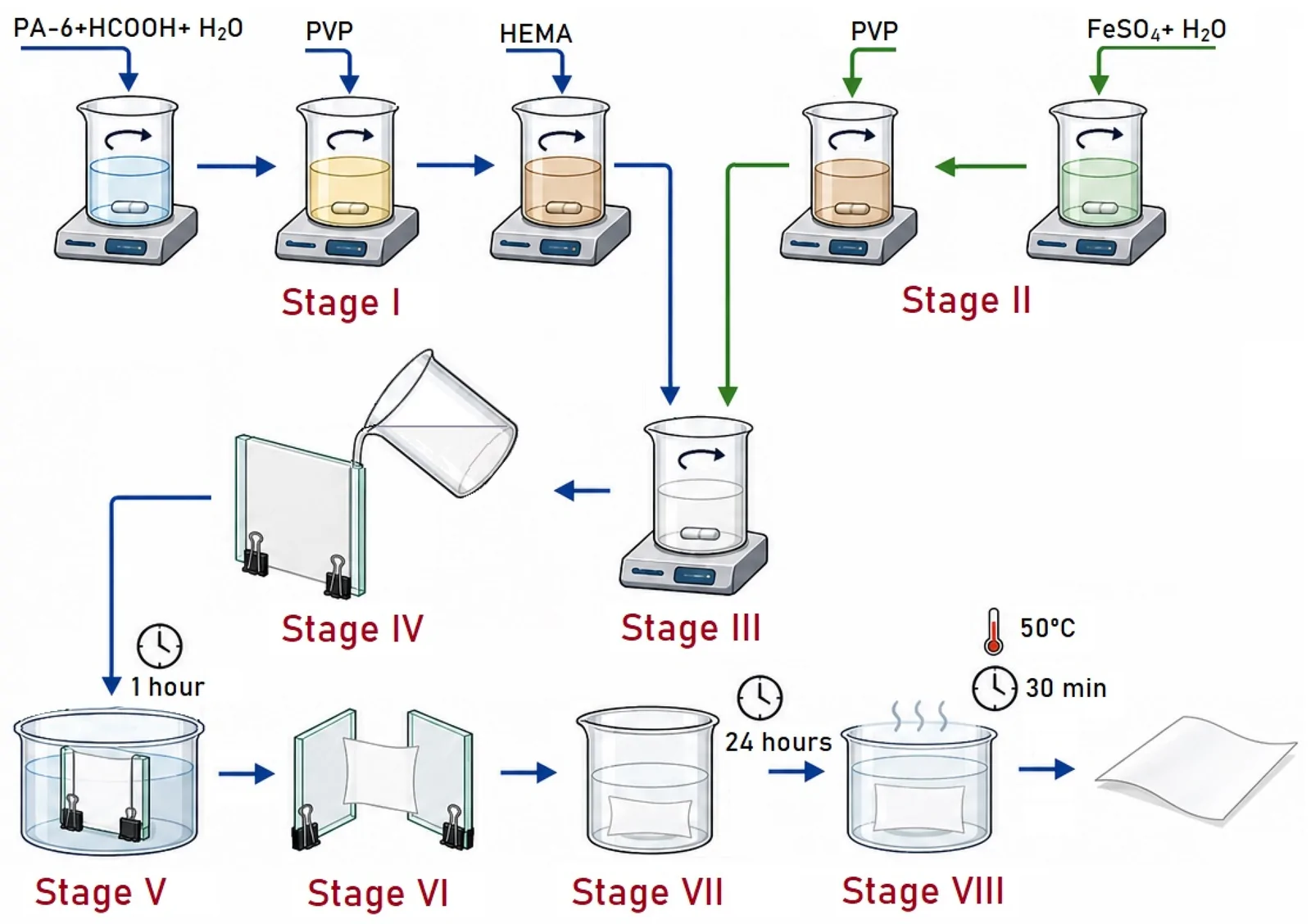
Schematic diagram of the preparation of composite hydrogel films based on PA-6/pHEMA-gr-PVP copolymers. Stages of the technological process: I—preparation of solution 1 (HEMA/PVP/H_2_O/PA-6/HCOOH); II—preparation of solution 2 (PVP/H_2_O/FeSO_4_); III—preparation of the reaction mixture; IV—dispensing the reaction mixture into the polymerization mold and polymerization; V—keeping the mold with the film in a water bath; VI—opening the mold and removing the film; VII—hydration of the film in distilled water; VIII—hydrothermal treatment. Generated using OpenAI, GPT-5.6 Luna based on authors prompts.

**Figure 1:**
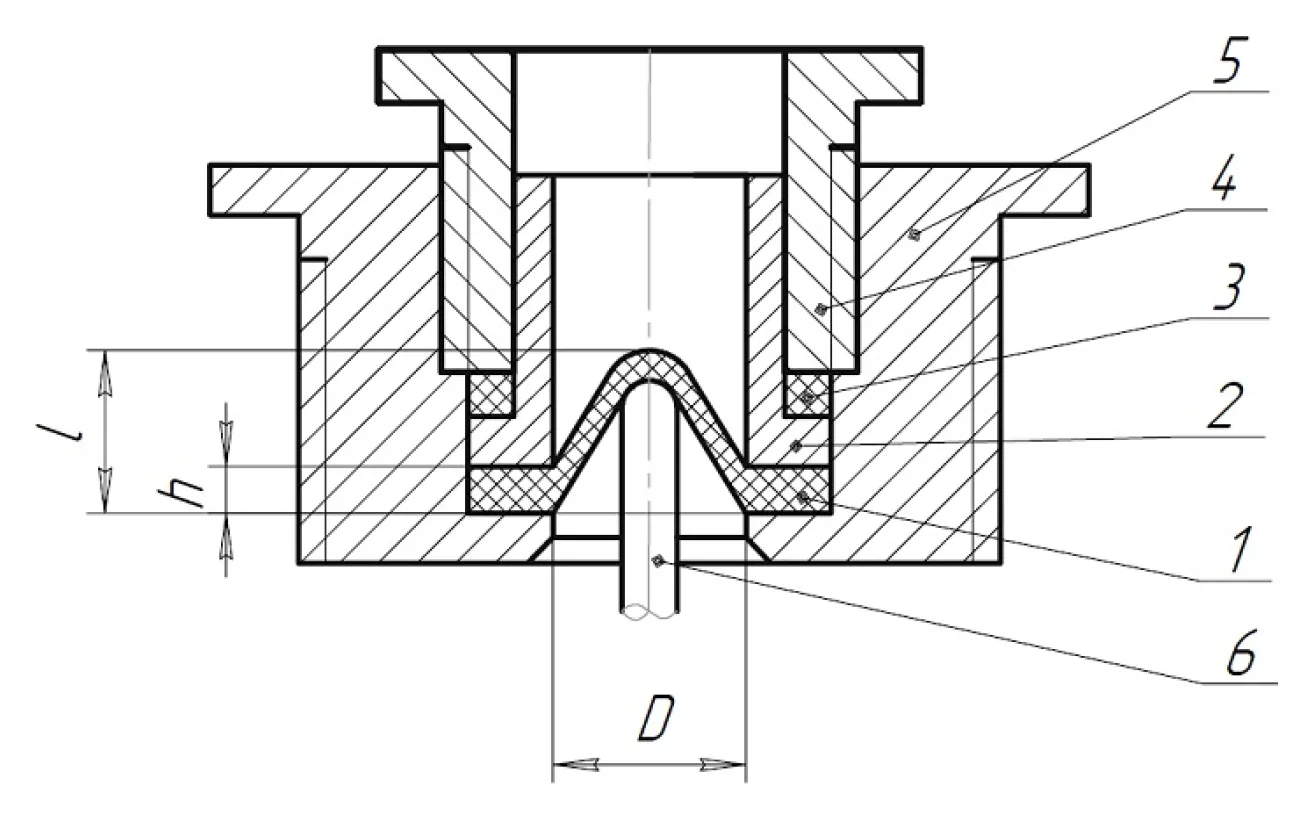
Schematic diagram of the device used to determine the physicomechanical properties of film materials by puncture: 1—film; 2—ring-shaped clamp; 3—fluoroplastic seal; 4—clamping nut; 5—holder; 6—pin.

**Figure 2:**
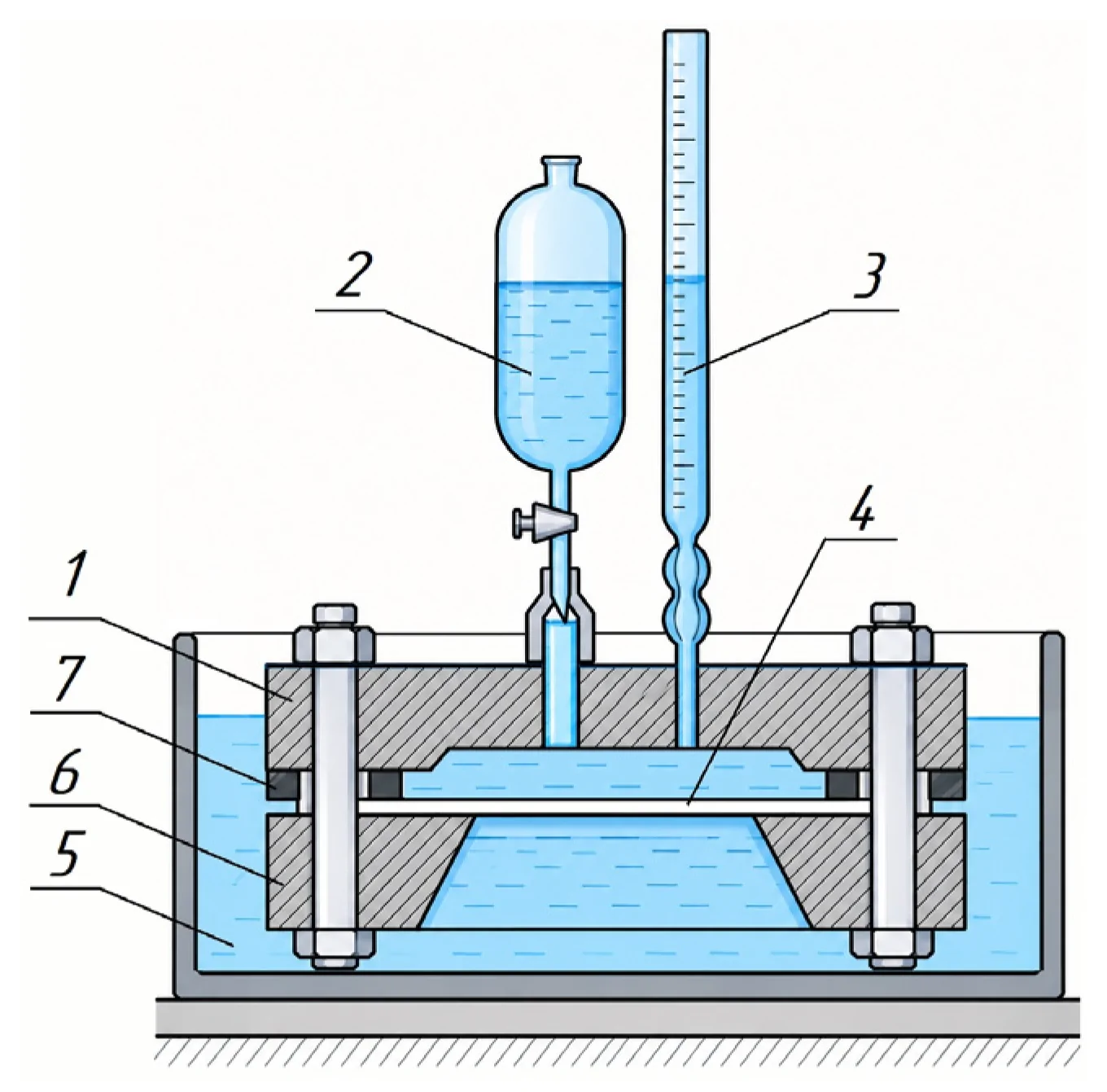
Schematic diagram of the device used to determine the osmotic permeability of hydrogel films: 1—osmometer lid; 2—salt solution vessel; 3—measuring capillary; 4—film; 5—water vessel; 6—osmometer body; 7—gasket. Generated using OpenAI, GPT-5.6 Luna, based on author prompts.

**Scheme 2:**
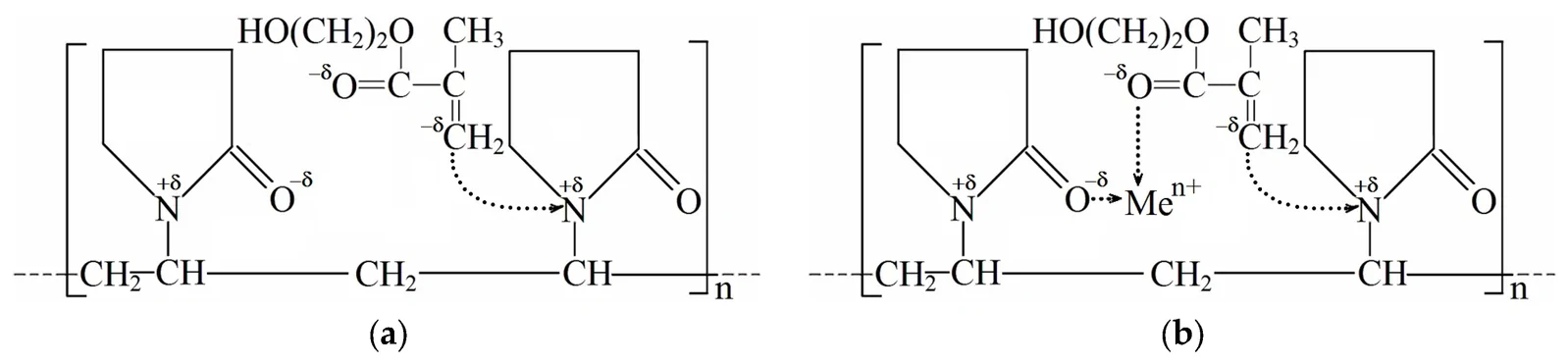
Charge transfer complexes: 2-hydroxyethyl methacrylate (HEMA)/polyvinylpyrrolidone (PVP) (**a**) and HEMA/Me^n+^/PVP (**b**) [34].

**Figure 3:**
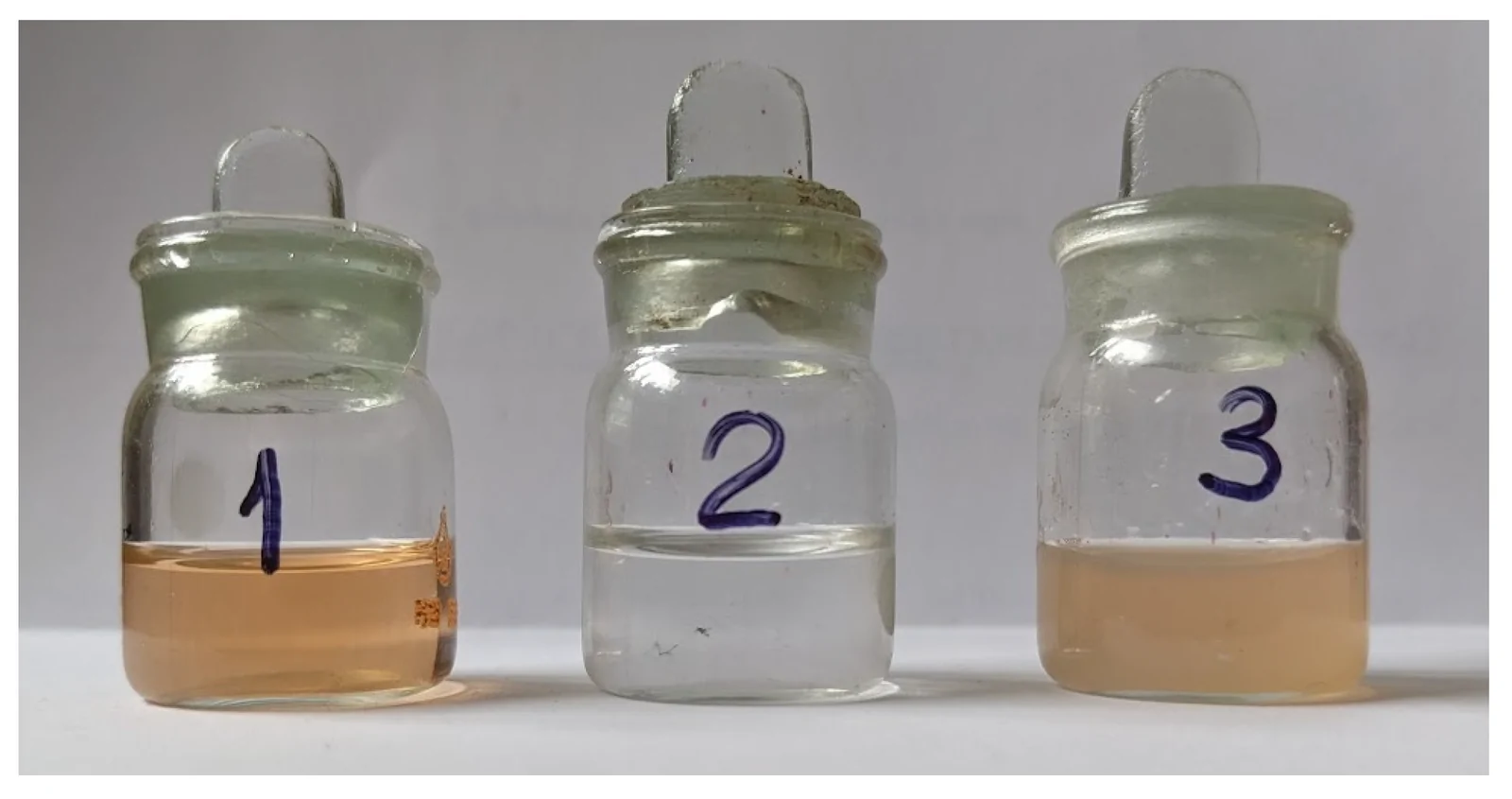
Solutions of PVP (1), PA-6 (2), and PA-6/PVP mixtures (3) in a water/formic acid solvent.

**Figure 4:**
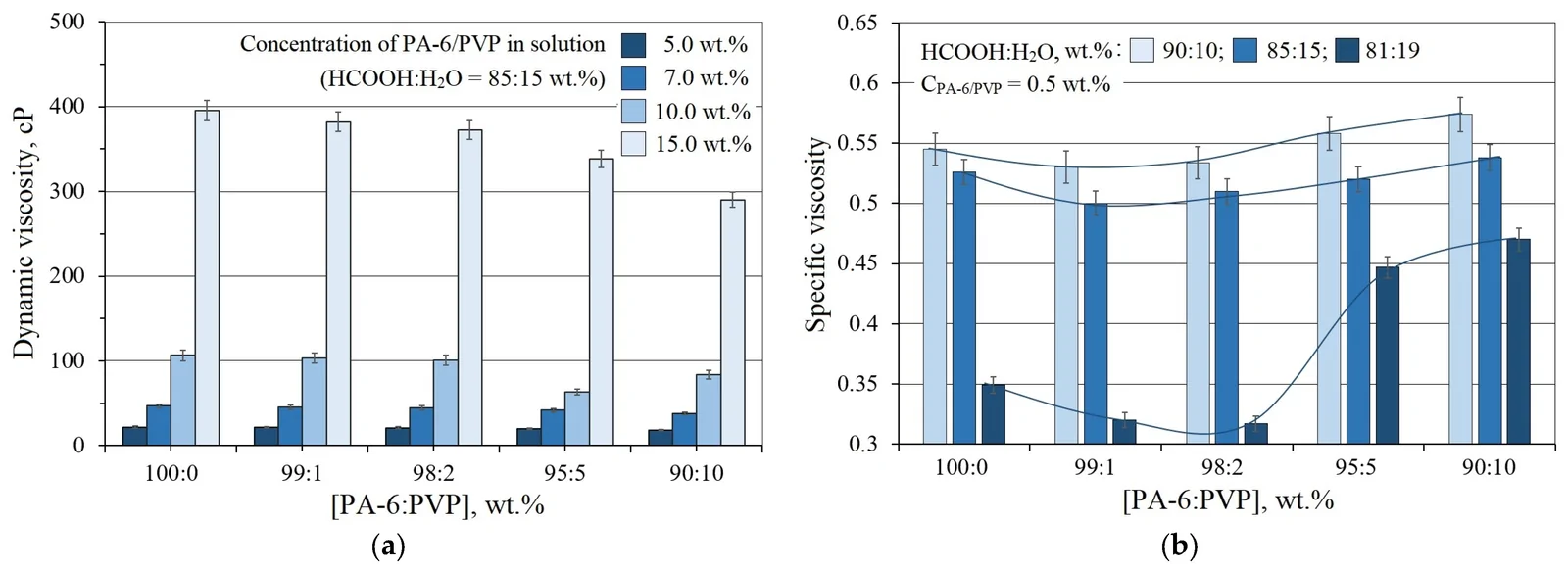
Dynamic (**a**) and specific (**b**) viscosities of solutions of the PA-6/PVP mixture in the formic acid/water solvent.

**Figure 5:**
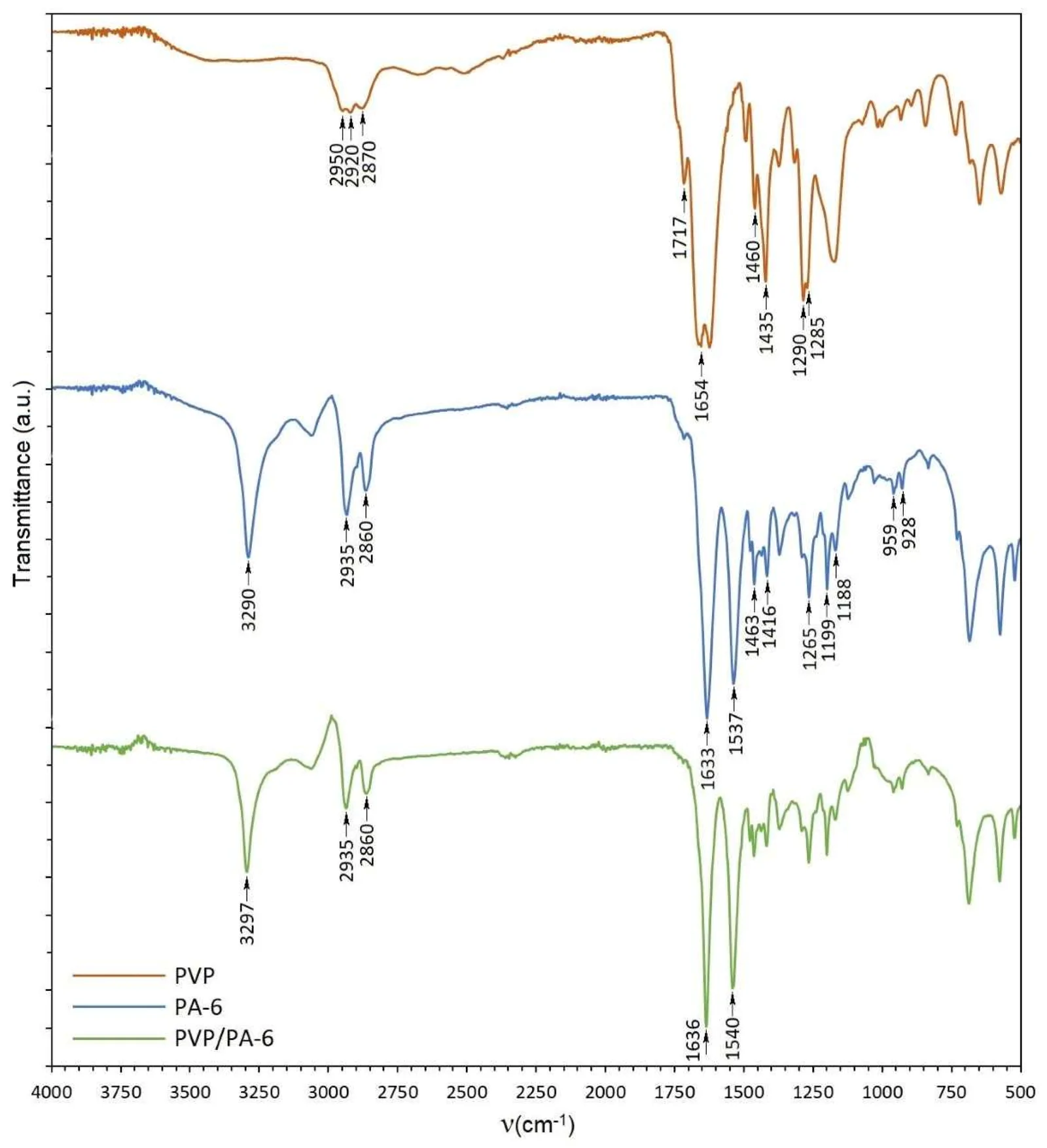
FTIR spectra of PVP, PA-6, and PVP/PA-6 mixtures.

**Figure 6:**
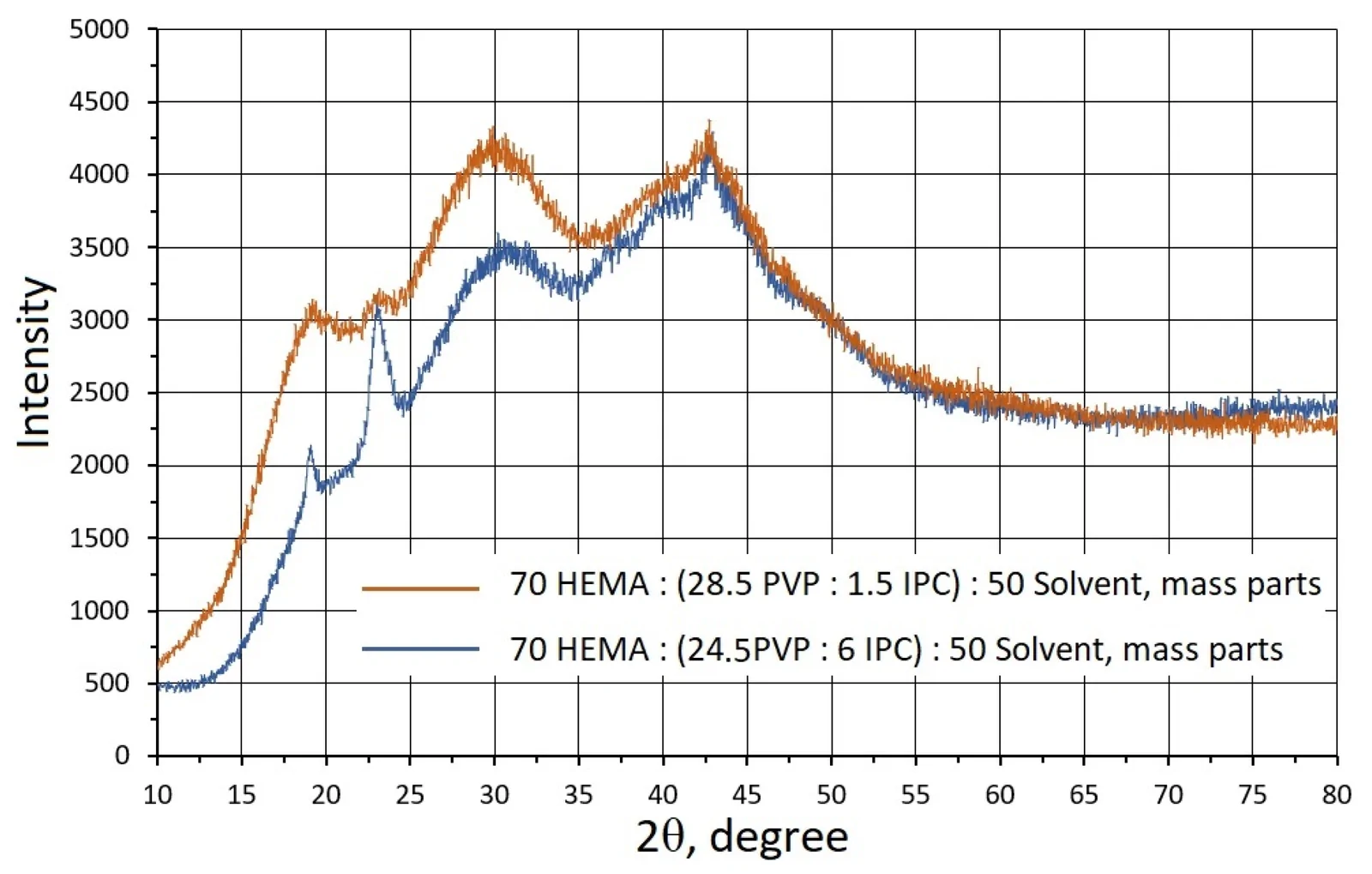
X-ray diffraction patterns of composite PA-6/pHEMA-gr-PVP copolymers with different polyamide contents in the IPC composition.

**Figure 7:**
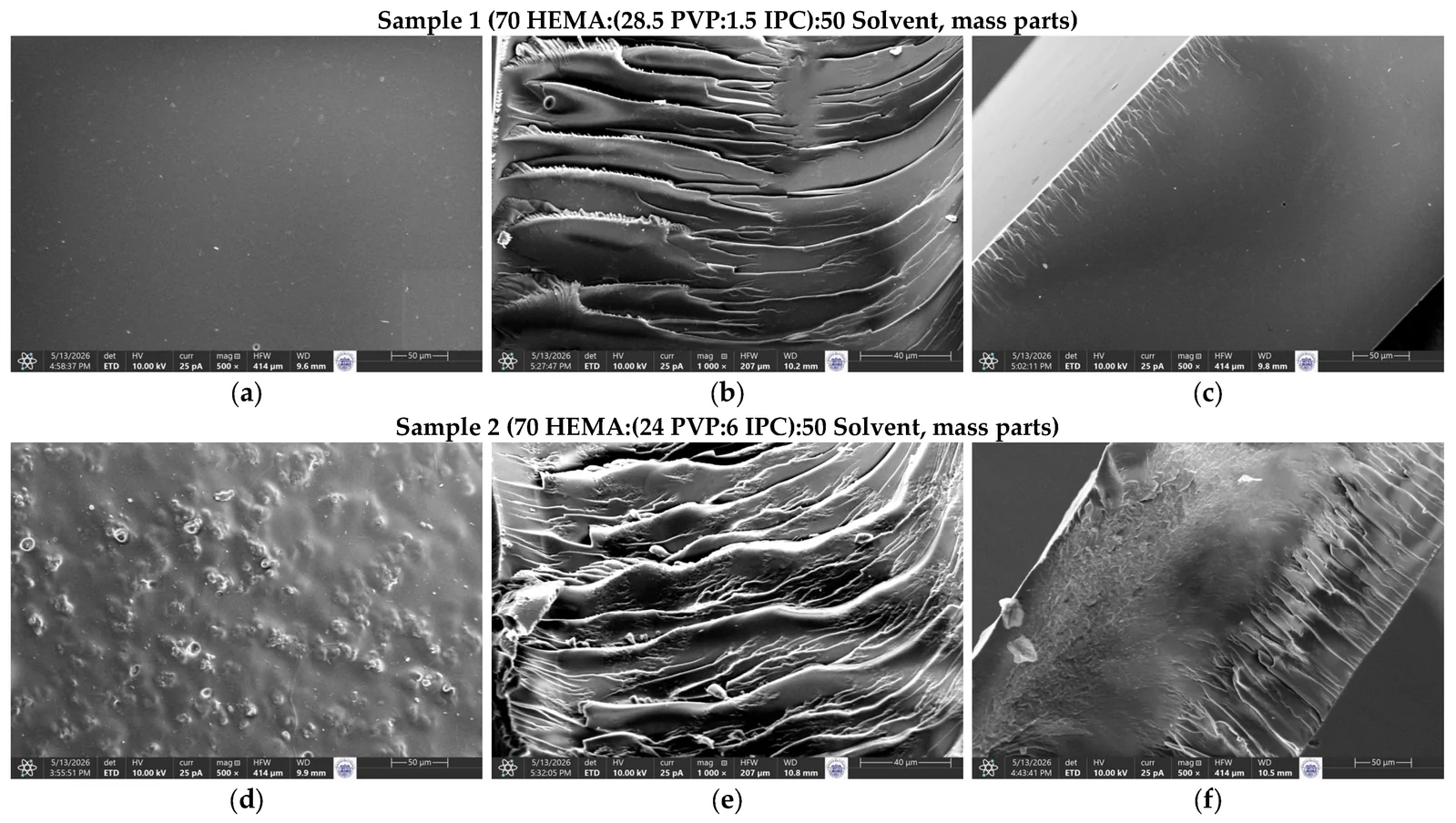
SEM images of the surface (**a**,**d**), fracture (**b**,**e**), and cross-sectional surface (**c**,**f**) of composite film samples based on PA-6/pHEMA-gr-PVP copolymers with different PA-6 content in the IPC.

**Figure 8:**
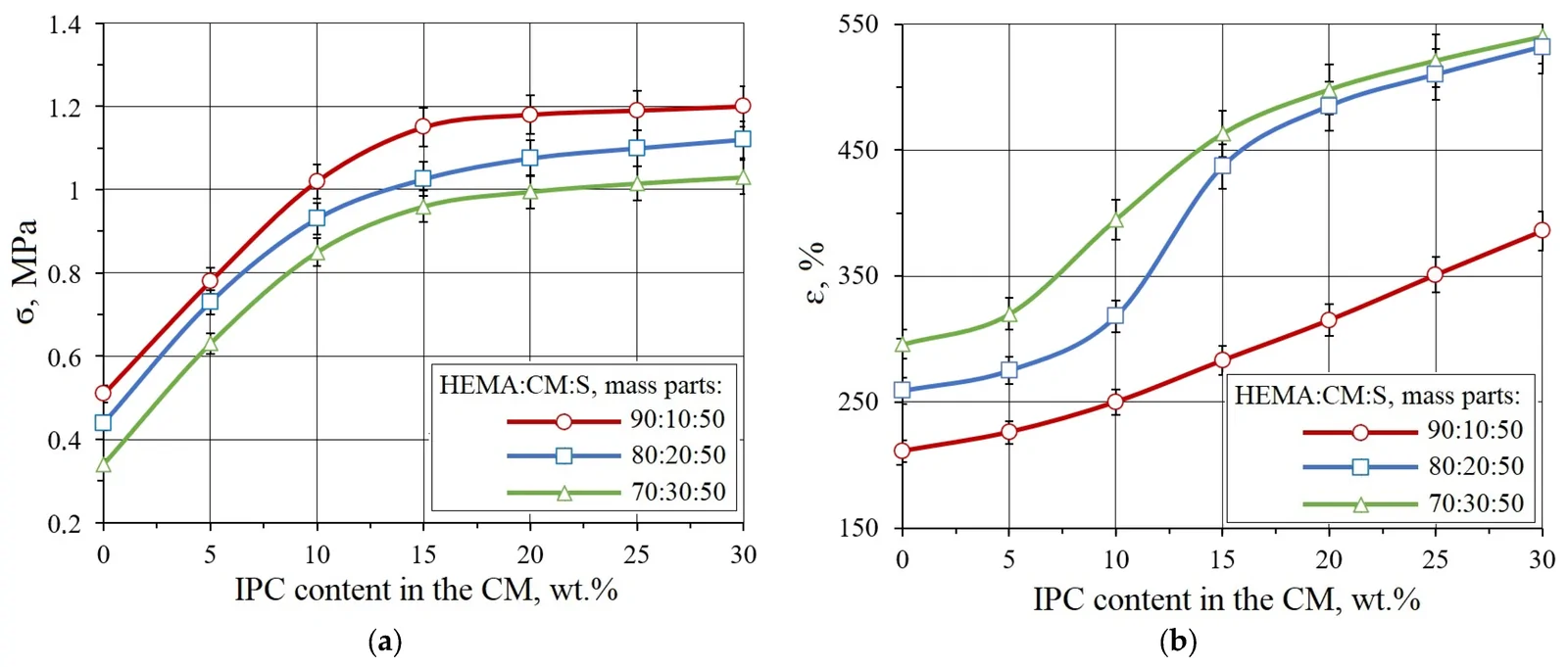
Effect of polymer–monomer composition (PMC) formula and interpolymer complex (IPC) content in the combined matrix (CM) on the puncture strength (**a**) and relative elongation at puncture (**b**) of composite hydrogel films.

**Figure 9:**
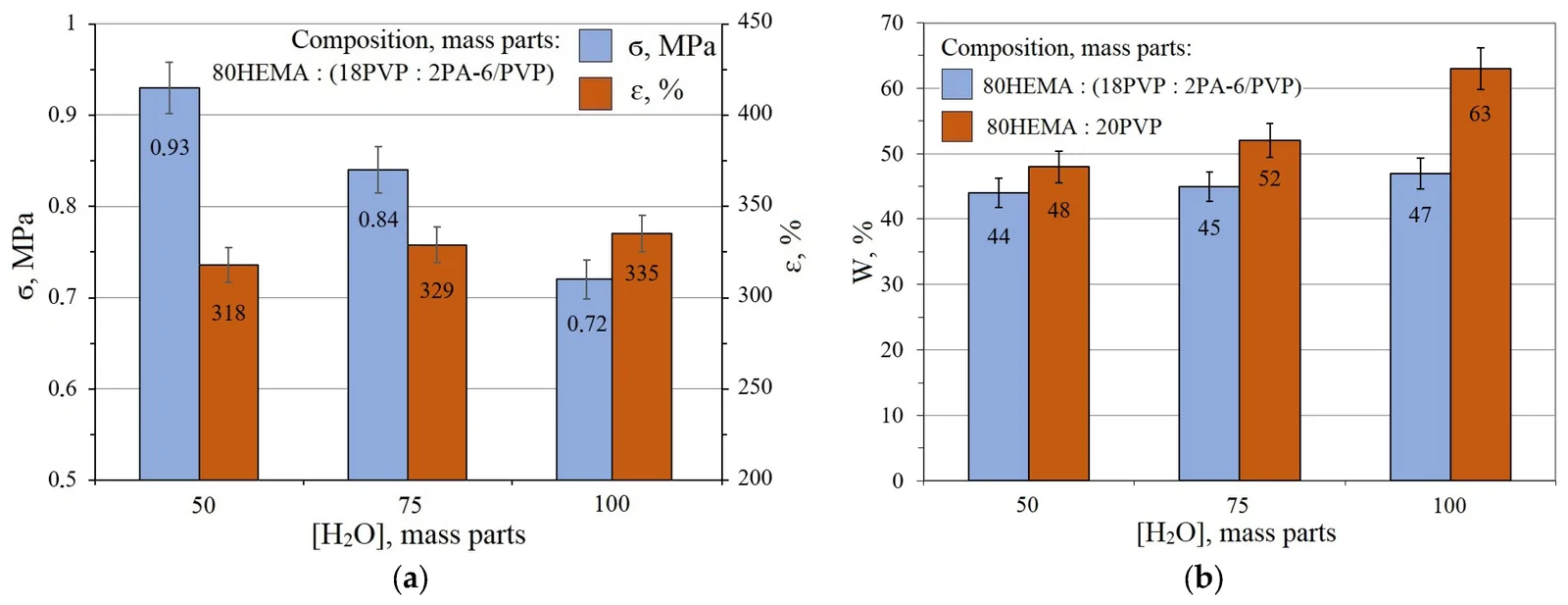
Effect of water content in the initial composition on the mechanical properties of the modified hydrogels, namely puncture strength and relative elongation at puncture (**a**), and on boundary water absorption (**b**).

**Figure 10:**
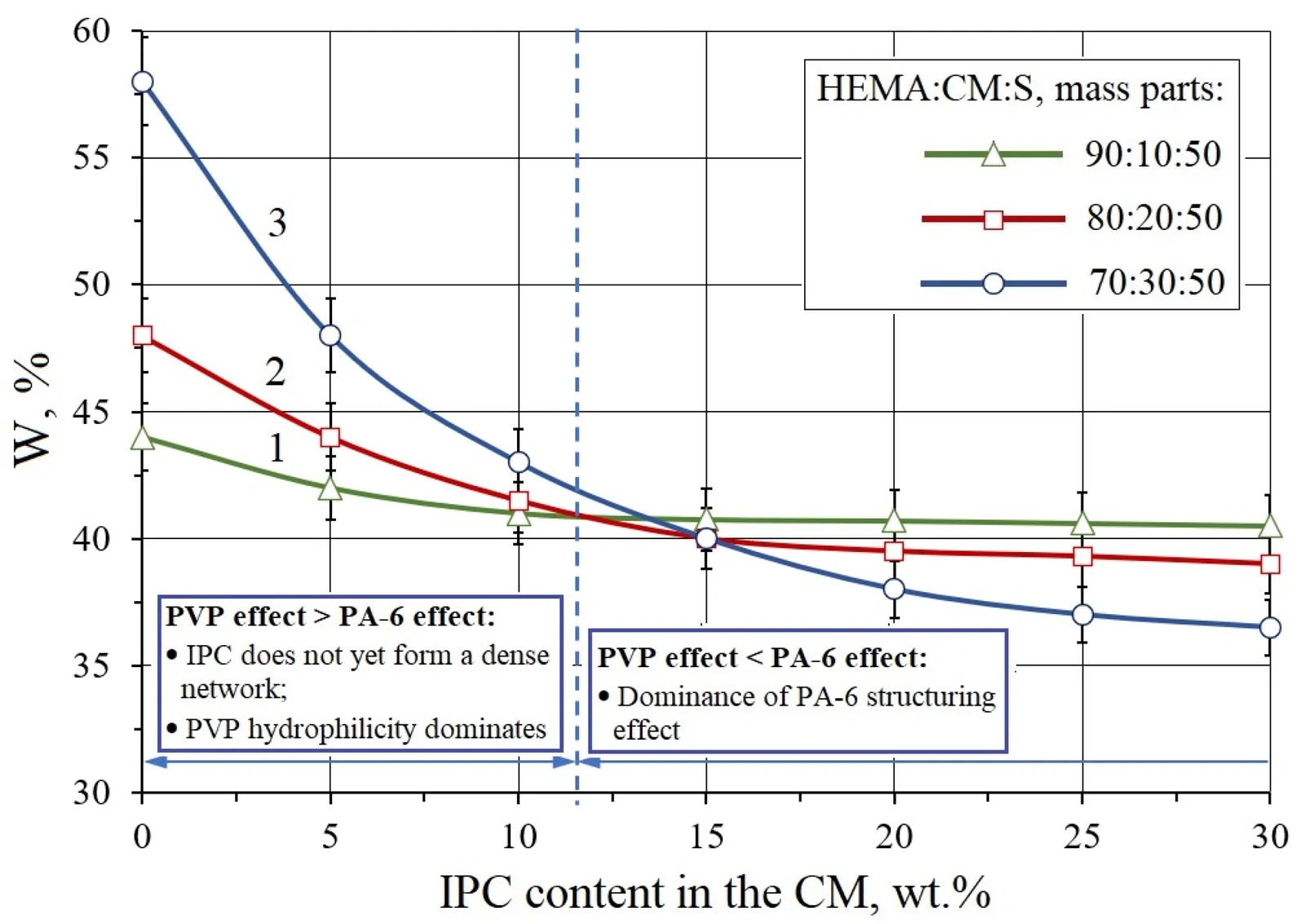
Effect of polymer–monomer composition (PMC) formula and interpolymer complex (IPC) content in the combined matrix (CM) on the water content of the modified hydrogels.

**Figure 11:**
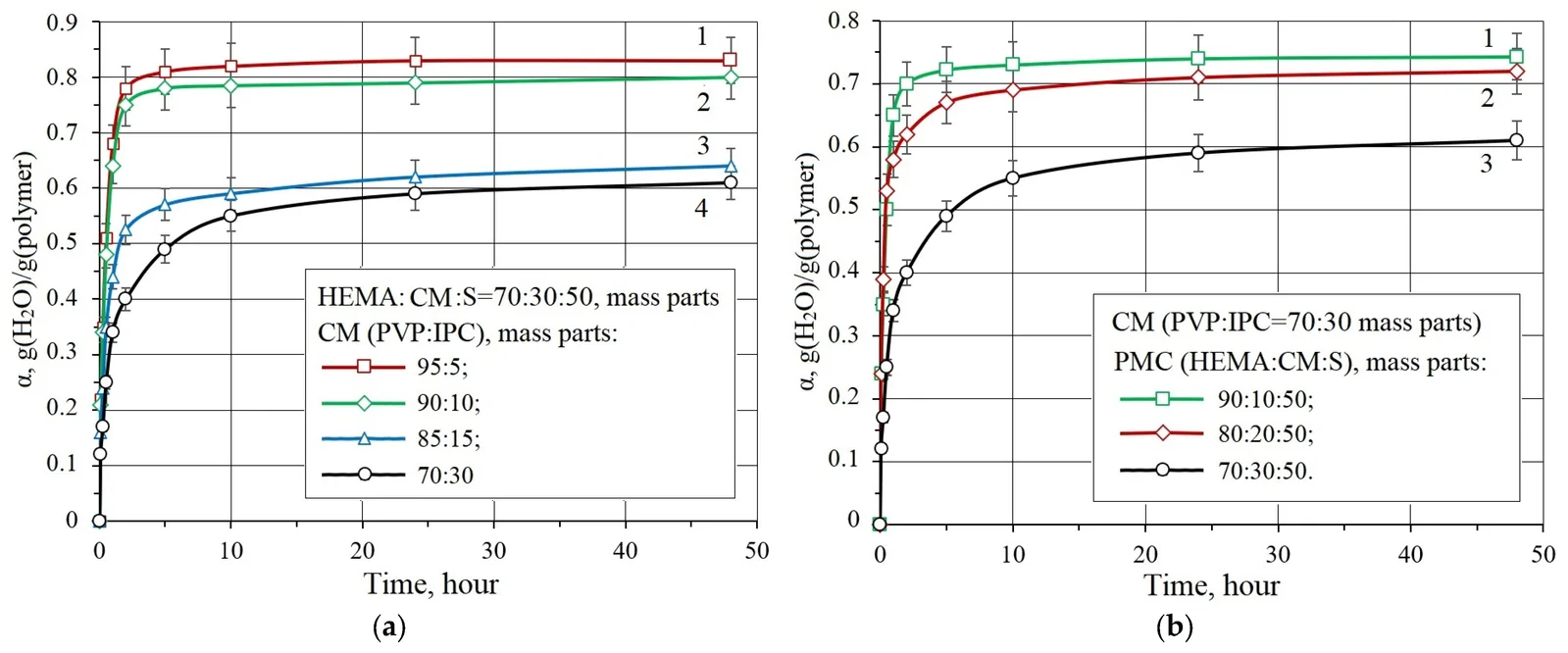
Effect of interpolymer complex (IPC) content (**a**) and polymer–monomer composition (PMC) formula (**b**) on the kinetic of changes in α of PA-6/pHEMA-gr-PVP copolymers.

**Figure 12:**
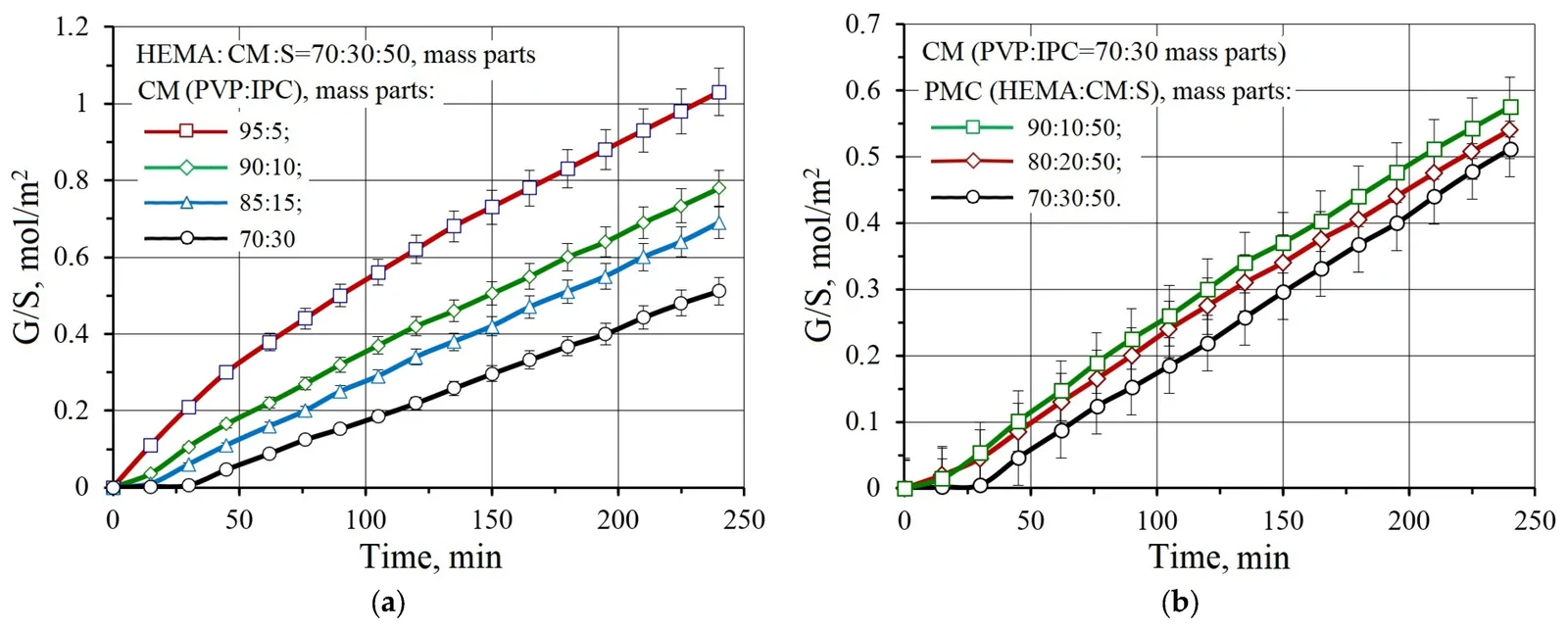
Effect of combined matrix (CM) content (**a**) and polymer–monomer composition (PMC) formula (**b**) on the permeability of PA-6/pHEMA-gr-PVP hydrogels.

**Table 1:** Composition of the Reaction Mixtures.

Reaction Mixture				
Polymer–Monomer Composition (PMC)			[Solvent] ^2^, Mass Parts	[FeSO_4_] ^3^, wt.%
[HEMA], Mass Parts	Combined Polymer Matrix (CM)			
	[PVP], Mass Parts/wt.%	[IPC] ^1^, Mass Parts/wt.%		
70	28.5/95	1.5/5	50	0.1
	27/90	3/10		
	25.5/85	4.5/15		
	24/70	6/30		
80	19/95	1/5	50	0.1
	18/90	2/10		
	17/85	3/15		
	14/70	6/30		
90	9.5/95	0.5/5	50	0.1
	9/90	1/10		
	8.5/85	1.5/15		
	7/70	3/30		

^1^ IPC—PA-6:PVP = 95:5 wt.% and IPC:HCOOH = 7:93 wt.% for all compositions. ^2^ Solvent—H_2_O + HCOOH. ^3^ [FeSO_4_] = 0.1 wt% of the PMC mass for all compositions.

## Data Availability

The data presented in this study are available on request from the corresponding author. The data are not publicly available due to privacy concerns.

## Abbreviations

The following abbreviations are used in this manuscript:

PVP	polyvinylpyrrolidone
HEMA	2-hydroxyethyl methacrylate
PA-6	polyamide-6
pHEMA-gr-PVP	copolymer of HEMA with PVP
IPC	interpolymer complex
pHEMA	PolyHEMA
CM	combined matrix
S	solvent
XRD	X-ray diffraction
FTIR	Fourier transform infrared
SEM	Scanning electron microscopy
PMC	polymer–monomer composition
CTC	charge-transfer complex
